# Generalized Hertz model for bimodal nanomechanical mapping

**DOI:** 10.3762/bjnano.7.89

**Published:** 2016-07-05

**Authors:** Aleksander Labuda, Marta Kocuń, Waiman Meinhold, Deron Walters, Roger Proksch

**Affiliations:** 1 Asylum Research, an Oxford Instruments company, Santa Barbara, CA, 93117, USA

**Keywords:** bimodal atomic force microscopy, bimodal spectroscopy, contact mechanics, multifrequency, nanomechanical mapping, nanomechanics

## Abstract

Bimodal atomic force microscopy uses a cantilever that is simultaneously driven at two of its eigenmodes (resonant modes). Parameters associated with both resonances can be measured and used to extract quantitative nanomechanical information about the sample surface. Driving the first eigenmode at a large amplitude and a higher eigenmode at a small amplitude simultaneously provides four independent observables that are sensitive to the tip–sample nanomechanical interaction parameters. To demonstrate this, a generalized theoretical framework for extracting nanomechanical sample properties from bimodal experiments is presented based on Hertzian contact mechanics. Three modes of operation for measuring cantilever parameters are considered: amplitude, phase, and frequency modulation. The experimental equivalence of all three modes is demonstrated on measurements of the second eigenmode parameters. The contact mechanics theory is then extended to power-law tip shape geometries, which is applied to analyze the experimental data and extract a shape and size of the tip interacting with a polystyrene surface.

## Introduction

Over the decades since its invention [1] the atomic force microscope (AFM) has been used in a variety of modes to characterize micro- and nanoscale heterogeneous structures in composites and other advanced materials. The AFM can provide high resolution topographic and mechanical properties mapping using techniques such as force curves [2–3], contact resonance [4–5], force modulation [6–7], phase imaging [8–9], loss tangent imaging [10], friction force microscopy [11], creep compliance [12], shear modulation force microscopy [13], pulsed force microscopy [14] and torsional approaches [15]. These techniques can be broadly classified as either “parametric” or “spectroscopic” techniques.

In parametric nanomechanical techniques, the sample properties are deduced from changes in the parameters of a driven cantilever that is oscillating in a (quasi) steady state while interacting with the sample surface. For example, tapping-mode AFM [16–17] (also known as amplitude-modulation (AM) AFM [18–20]), is one of the most commonly used parametric techniques, where the cantilever is driven on resonance and the cantilever–sample distance is adjusted by a feedback loop to maintain a constant oscillation amplitude at every image pixel. The time required for the cantilever to reach a steady state defines the acquisition speed, allowing tapping-mode imaging to achieve very high speeds ultimately only limited by the cantilever bandwidth. However, the small number of tapping-mode observables (amplitude and phase) limits the extraction of absolute storage and loss moduli, as they cannot be distinguished from changes in indentation depth. In tapping mode, only the *ratio* of the storage to loss modulus can be measured [10,21–22]. The same limitation applies to many other parametric techniques, such as force modulation [6–7] and other single-frequency imaging modes, such as frequency-modulation (FM) AFM [23]. Separating the storage and loss moduli, and quantifying them, requires either additional independent observables or the use of spectroscopic methods.

Spectroscopic techniques rely on changing the operating conditions of the cantilever to provide the necessary information to extract nanomechanical properties of the sample for a given image pixel. This can be achieved by changing the cantilever–sample distance [24] or sweeping the drive frequency [25], amongst others [26]. Examples of well-established spectroscopic techniques are nanoindentation [27] and force curves as well as dynamic force curves performed with an oscillated cantilever. The time-varying cantilever response serves as input to a model for extracting nanomechanical properties of the sample at any location. These techniques are by nature slow for imaging, as they measure time-varying changes of the cantilever at every pixel.

Recently, parametric techniques have been extended by driving two or more cantilever resonances simultaneously in order to increase the number of observables, which is required to quantify the storage and loss moduli. Advances in this direction include bimodal [28–33], trimodal [34] or more generally multimodal/multifrequency [35] techniques, and have demonstrated quantitative mapping without compromising on the high speeds that define parametric imaging techniques. Currently, state-of-the-art bimodal methodologies are mostly based on FM-AFM techniques that rely on elaborate mathematical theories [36–41], involving fractional calculus and Laplace transforms for relating AFM observables to nanomechanical properties. The mathematical complexity of these techniques can obscure physically intuitive understanding of the cantilever dynamics in bimodal AFM experiments.

**Figure 1:**
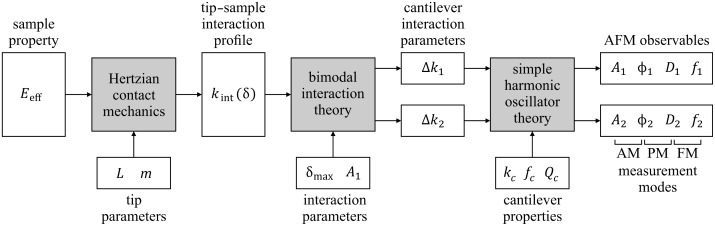
Diagram of the theory presented in this paper, showing relationship between inherent sample properties and tip geometry parameters to the AFM observables for bimodal AFM in the context of Hertzian contact mechanics. The tip shape is assumed to have a power-law profile with size parameter *L* and shape parameter *m*.

Here, we present a simplified theory for bimodal AFM with a *large* fundamental resonance oscillation amplitude and *small* higher resonance amplitude. The theory is based on a binomial approximation of the weight function for extracting the interaction stiffness for both resonant modes, each yielding a simple analytical expression. These two independent pieces of information are refactored to provide information about modulus and indentation depth. While the theory is generally applicable to a wide range of tip–sample interaction models, the derivation here begins in the context of a Hertzian contact with a paraboloidal tip and is then generalized to any tip shape described by a power-law profile. This theory is then experimentally applied to three variations of bimodal AFM involving different dynamic AFM modes of operation [42–43], namely amplitude modulation (AM) [1,18–19], phase modulation (PM) [43–46] and frequency modulation (FM) [23,36,47]. Finally, a method for extracting the tip size and tip shape from bimodal AFM approach curves is presented and demonstrated on a polystyrene sample. Figure 1 provides a diagram of the theory presented in the following three sections.

## Methods

### Hertzian contact mechanics

The Hertzian contact model involves the interaction stiffness *k*_int_ versus indentation depth δ between a paraboloidal tip of radius *R* and a flat sample as

[1]
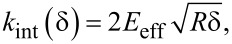


where the effective Young’s modulus *E*_eff_ combines deformation of the tip and sample [48]. Similar expressions can be derived for a tip in the shape of a punch or a cone [49].

Here, these three special cases are generalized to any axisymmetric tip shape whose cross-sectional radius *r*, as a function of height *z*, is governed by the power law

**Figure 2:**
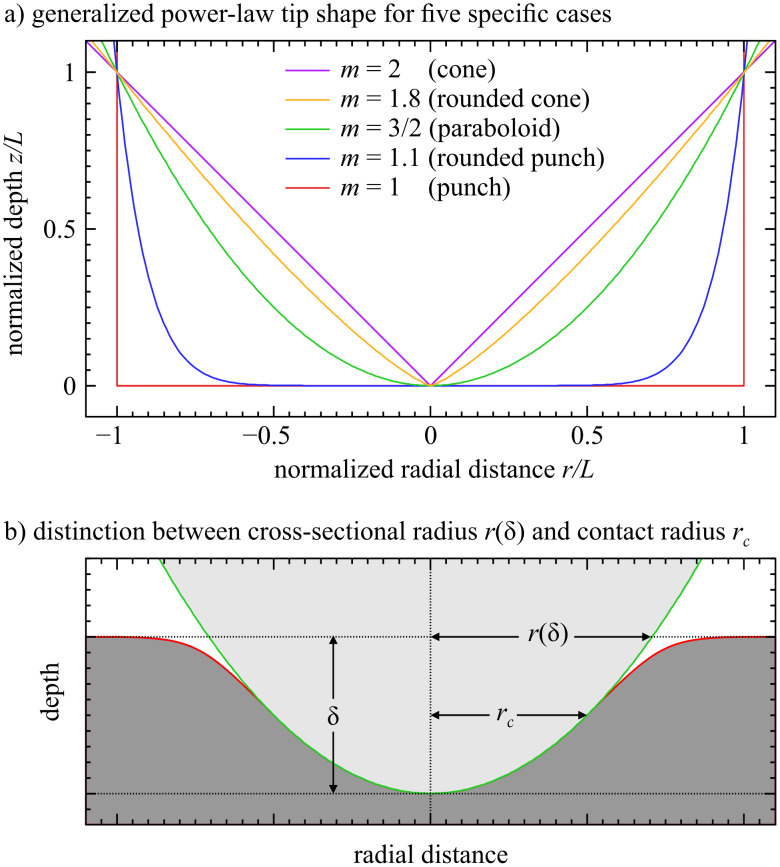
a) The generalized tip shape is drawn for various values of *m*. b) The indentation of a paraboloidal indenter into an elastic surface illustrates the distinction between the cross-sectional indentation radius versus the true contact radius.

[2]
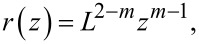


where the characteristic length scale *L* and the exponent parameter 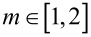 fully define the tip size and shape, respectively. Figure 2a illustrates Equation 2 for five values of *m*, including the three special cases of punch, paraboloid, and cone. Note that “sphere” is used as a shorthand for “paraboloid” in the scientific vernacular.

Upon purely elastic indentation of such a power-law indenter into a sample surface to an indentation depth δ, the true contact radius *r*_c_ is smaller than the cross-sectional radius *r*(δ) because of deformation of the surface, as can be understood from Figure 2b. The exception is the punch model for *m* = 1, where no reduction in radius occurs. The contact radius correction factor α_c_ quantifies the reduction in contact radius *r*_c_ with respect to *r*(δ) by

[3]
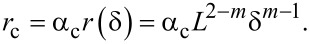


As expected, α_c_ = 1 for *m* = 1 (punch), and then α_c_ monotonically decreases to α_c_ = 2/π ≈ 0.64 for *m* = 2 (cone). The mathematical form of α_c_ is presented in the Appendix (b).

Importantly, it is the true contact radius *r*_c_ that defines the tip–sample interaction stiffness, as derived by Oliver and Pharr [27]:

[4]
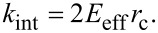


Substituting in the expression for *r*_c_ leads to the general form

[5]
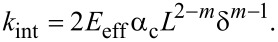


**Figure 3:**
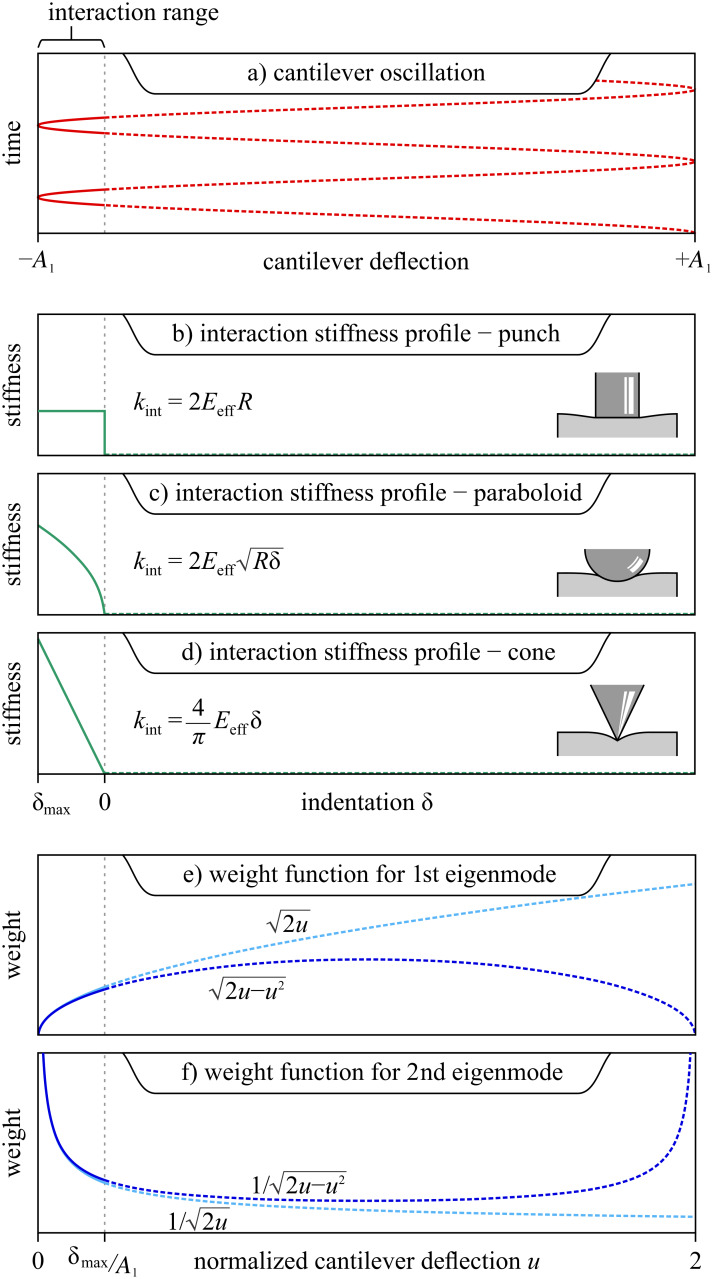
a) The cantilever motion during oscillation in bimodal imaging with only a small portion interacting with the sample. Note that the oscillation of the second eigenmode cannot be seen, as it is assumed infinitely small by the model proposed here. The stiffness profile of the interacting tip is plotted for three tip shapes: b) punch, c) paraboloid, d) cone. The weight functions used for integrating these stiffness profiles are shown for e) the first eigenmode (assuming a large amplitude) and f) the second eigenmode (assuming a small amplitude). The weight function for the second eigenmode diverges where the interaction stiffness is nonzero, reflecting the sensitivity of bimodal AFM to nanomechanical properties. The 
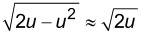
 binomial approximation is illustrated for both weight functions; they are very accurate approximations in the interaction range, and can be used to derive a simple analytical solution for bimodal imaging. Note: the three different *x*-axes shown in these plots are interchangeable; they reflect the most appropriate parameterization of the tip position in each case.

The interaction stiffness in Equation 5 is plotted for three special cases in Figure 3. For the punch model with *m* = 1, the length scale parameter *L* is equal to the punch radius *R*. For the paraboloidal indenter model with *m* = 3/2, *L* is the effective “sphere” diameter 2*R*. For the conical indenter with *m* = 2, *L* drops out and the cone half-angle θ = 45°. (Given the loss of the length scale parameter *L* in the degenerate case *m* = 2 (cone), a half-angle parameter θ may be introduced by multiplying *k*_int_ by (tan θ)^−1^ to fully define the geometry of the conical indenter, if necessary.)

### Bimodal interaction theory

This section first describes how the tip–sample interaction stiffness of a paraboloidal (“spherical”) tip affects the changes in effective stiffness of the first and second eigenmodes of the cantilever. Then, the results are generalized to a power-law tip shape.

#### Paraboloidal tip

For the first eigenmode driven with a *large* oscillation amplitude *A*_1_, the change in stiffness of the interacting cantilever Δ*k*_1_ averaged over one cycle can be computed by integrating *k*_int_(δ) with a semi-circular weight function that spans the peak-to-peak cantilever oscillation [50], as represented in Figure 3. Mathematically,

[6]
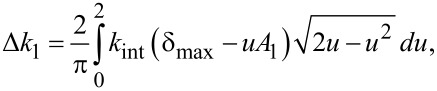


where δ_max_ is the maximum indentation depth and the normalized distance *u* = (δ_max_ − δ)/*A*_1_. It is worth noting the distinction between *k*_int_(δ), which is the instantaneous stiffness *profile* experienced by the oscillating cantilever tip, and Δ*k*_1_, which is the time-averaged effective change in stiffness of the cantilever–tip–sample system that is experimentally measurable by the AFM user. Measuring Δ*k*_1_ is the topic of Section ‘Simple harmonic oscillator theory‘, presented later.

In the limit that the fundamental amplitude *A*_1_ is much larger than the interaction length scale, the stiffness profile *k*_int_(δ) only affects a small portion of the cantilever sinusoidal oscillation where the tip indents the sample, as shown in Figure 3a. Therefore, the integration limits in Equation 6 reduce to [0, δ_max_/*A*_1_] such that *u* << 1 throughout the integration. Consequently, the weight function can be approximated very accurately with the first term in the binomial expansion, 
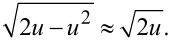
 This approximation, used previously [51], is graphically illustrated in Figure 3e,f. Applying it to Equation 6 results in

[7]
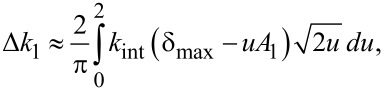


which can be easily integrated for a paraboloidal indenter to give

[8]
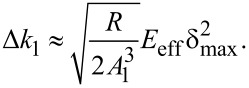


**Figure 4:**
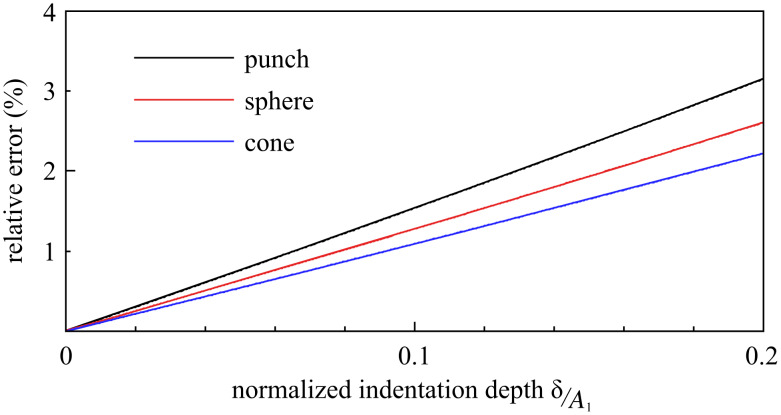
The relative error introduced in the calculation of Δ*k*_1_ because of the 
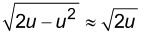
 approximation applied to Equation 6 in the case of a punch, paraboloid, and cone contact model. The 
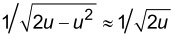
 approximation applied to Equation 9 results in a similar, albeit negative, error.

The relative error in Δ*k*_1_ introduced by the binomial approximation is quantified in Figure 4; it typically falls on the order of 1% in large-amplitude dynamic AFM, where the interaction amplitude *A*_1_ greatly exceeds δ_max_.

Meanwhile, the second (or higher) eigenmode is deliberately driven at a *small* amplitude *A*_2_, such that the interaction stiffness it experiences is roughly constant throughout one of the higher eigenmode oscillation cycles. However, the instantaneous interaction stiffness experienced by the second eigenmode slowly changes along the trajectory of the first eigenmode. Because the second mode rides along the slower sinusoidal motion of the first mode, its time-averaged change in interaction stiffness Δ*k*_2_ can be calculated by

[10]
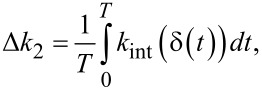


where *T* is the oscillation period of the first eigenmode. A rigorous derivation of Equation 10 is provided in Appendix (a). Parametrizing this time integral with respect to distance by the substitution *t* = cos^−1^(*u*)/ω_1_ results in

[9]
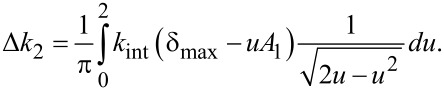


Similarly to the approach taken with the first eigenmode, this integral can be solved analytically for a paraboloidal indenter after applying the binomial approximation 
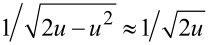
 such that

[11]
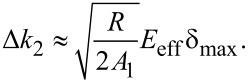


Note that the second-mode change in stiffness scales linearly with indentation depth, Δ*k*_2_
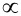
δ_max_, while the first mode stiffness scales with the square of the indentation depth, 
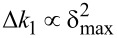
. These scaling laws have been verified experimentally for a paraboloidal indenter [39]*.* The system of two equations (Equation 8 and Equation 11) can be solved for two unknowns, namely

[12]
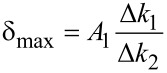


and

[13]
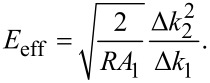


This operation is central to bimodal imaging, as it separates the changes in modulus from changes in indentation depth. This distinction cannot be made in single-mode dynamic AFM imaging.

The key to bimodal nanomechanical imaging is that the *same* stiffness profile is measured simultaneously by two different eigenmodes with different weight functions: 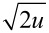 and 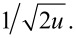 The fact that these weight functions are related by a derivative operation results in independent measures of nanomechanical properties by both eigenmodes while imaging. Notably, the fact that the weight function of the second eigenmode increases drastically as the tip approaches the sample, as seen in Figure 3f, explains the high sensitivity and increased spatial resolution of bimodal imaging noted in the past [52].

#### Power-law tip

The derivation so far revolved about a paraboloidal indenter. Applying the same approach used in Equations 6–13 to the generalized stiffness profile of Equation 5 result in the generalized indentation and modulus equations

[14]
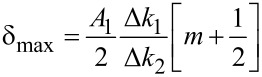


and

[15]
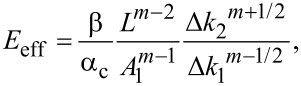


where β is a scaling constant that depends only on *m* and is mathematically defined in the Appendix (c). As expected, choosing *m* = 3/2 in Equation 14 and Equation 15 recovers Eqution 12 and Equation 13.

The tip shape exponent *m* is a free parameter in the generalized stiffness profile. It can be inferred experimentally by measuring changes in Δ*k*_1_ and Δ*k*_2_ for a fixed *A*_1_ and relating them to Equation 15 by the scaling law

[16]
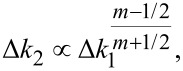


as will be demonstrated later.

### Simple harmonic oscillator theory

Measurements of Δ*k*_1_ and Δ*k*_2_ are necessary for calculating δ_max_ and *E*_eff_ in Equation 12 and Equation 13 or Equation 14 and Equation 15.

A single equation that extracts Δ*k* from a driven eigenmode (on and off resonance) with any number of changing AFM observables is presented in the Appendix (d). The derivation assumes the eigenmode in question can be modeled as a simple harmonic oscillator (SHO) within the range of interactions explored in the experiment. Here, three special cases (AM, PM, FM) for starting an experiment on-resonance were extracted from the general SHO solution for simplicity. These special cases are presented in Table 1, and described in the following subsections. The constant-excitation FM mode (CEFM) is also included in Table 1 for completeness.

**Table 1:** Using an automatic-gain-controller (AGC) and/or phase-locked-loop (PLL) to control the cantilever amplitude and frequency, respectively, leads to four combinations of measurement modes: amplitude modulation (AM), phase modulation (PM), frequency modulation (FM), and constant-excitation frequency modulation (CEFM). Using these modes for measuring Δ*k*_1_ and Δ*k*_2_ of a cantilever independently leads to 16 bimodal configurations: AM-AM, AM-PM, AM-FM, FM-FM, etc.

		Phase-locked-loop (PLL)	
		OFF	ON

Automatic-Gain-Controller (AGC)	OFF	AM mode	CEFM mode
	ON	PM mode	FM mode

The last subsection extends the SHO theory to measuring two eigenmodes simultaneously, as required for bimodal AFM.

#### Amplitude modulation (AM) mode

For an eigenmode driven in AM mode, the corresponding time-averaged interaction stiffness can be calculated from the measured interaction amplitude *A* and interaction phase 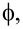 as in

[17]
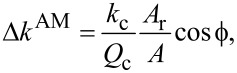


while the cantilever quality factor *Q*_c_, the cantilever stiffness *k*_c_, and the reference amplitude *A*_r_ are all measured in the absence of tip–sample interactions (*A*_r_ is often referred to as the “free amplitude”).

#### Phase modulation (PM) mode

Alternatively, PM mode uses an automatic-gain-controller (AGC) to maintain a constant cantilever amplitude *A* by varying the drive amplitude *D*; meanwhile, the drive frequency remains fixed just as in AM mode. In this mode,

[18]
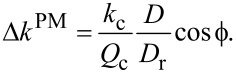


Note that an AGC is especially beneficial for use on the second eigenmode, because *A*_2_ is often chosen close to the detection limit and may drop substantially in AM mode when the resonance frequency shifts upon interaction with the sample. The AGC in the PM mode assures that *A*_2_ remains above the detection limit throughout the experiment.

#### Frequency modulation (FM) mode

If FM mode is employed for any of the eigenmodes, where the resonance frequency *f*_c_ is tracked with a phase-locked-loop (PLL), the measured frequency shift Δ*f* can be used to estimate the interaction stiffness by the approximation

[19]
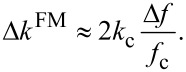


In this mode, the oscillation amplitude is held constant with an AGC. The use of an AGC will be assumed for “FM mode” henceforth.

Alternatively, constant-excitation FM mode (CEFM) employs a PLL but does not use an AGC [53–54]; therefore, the oscillation amplitude is not necessarily constant throughout the tip–sample interaction. This less-common technique is not explored in this article.

#### Bimodal configurations

In bimodal AFM, both eigenmodes behave like independent SHO’s because the first mode is driven in the large-amplitude limit and the second is driven in the small-amplitude limit. An empirical verification of this independence will be performed and discussed later.

In principle, both Δ*k*_1_ and Δ*k*_2_ can be measured with any of the four measurement modes described so far, leading to 16 possible configurations: AM-AM, AM-PM, AM-FM, FM-FM, etc. As an example, the first experimental bimodal measurements were taken in the AM-AM configuration [33]. In their pioneering work, Heruzzo and Garcia demonstrated FM-FM measurements [39,55]. Here, the AM-AM, AM-PM and AM-FM configurations are explored in detail. Practical considerations for these choices will be discussed later.

## Results

A polystyrene surface was probed with bimodal approach curves (also known as “force–distance” curves), where the bimodally oscillating cantilever approaches the sample with a constant velocity. The experiment is described in the first subsection, where the AM-AM, AM-PM, and AM-FM configurations are compared to investigate the validity of the SHO model assumed by the bimodal theory. Then, multiple approach curves are used to extract the shape and size of the tip. Finally, the newly defined tip geometry is used to extract the modulus from all the approach curves to assess consistency of the results and their dependence on imaging parameters.

### Experiment

The experiment was performed on a Cypher AFM with an ASYELEC-02 cantilever, which has a Ti/Ir-coated tip of nominal tip radius *R* = 28 ± 10 nm. Photothermal excitation [56] was used, which ensures stable imaging [57] and accurate FM tracking [58–60]. An automated calibration method [61] was used to obtain the stiffness of the first eigenmode (*k*_c1_ = 43.2 N/m), which was then used as a basis of calibration for the second eigenmode stiffness *k*_c2_ = 818 N/m by a recently established calibration protocol [62]. Next, a thermal power spectral density [63] of the cantilever was acquired close to the surface (within a few hundred nanometers) to determine the remaining cantilever properties (*f*_c1_, *f*_c2_, *Q*_c1_, *Q*_c2_). For this calibration step, the proximity to the surface is important. Performing it far from the surface incorrectly introduces long-range cantilever–sample interactions into the final measurement. Finally, the equipartition theorem was used to convert the amplitudes of both eigenmodes into nanometers [64–66].

**Figure 5:**
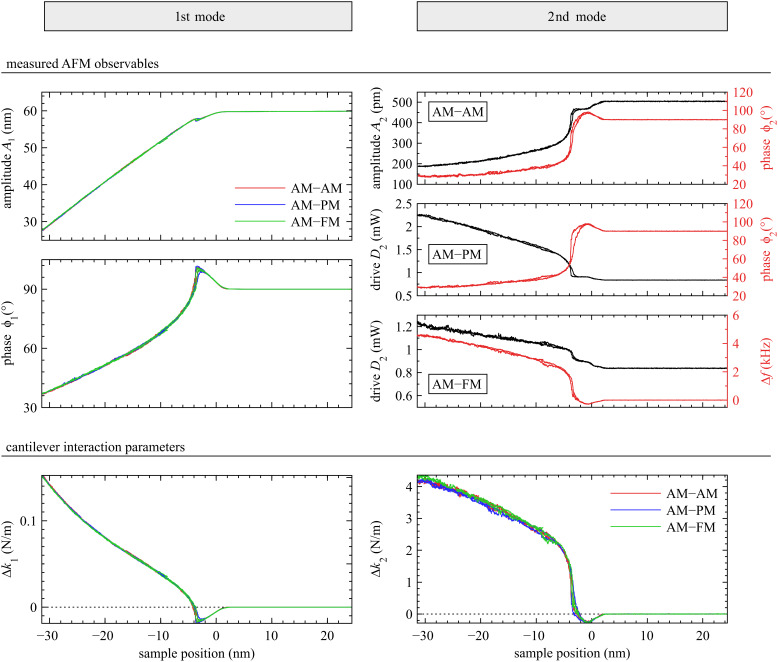
Experimental results from three consecutive approach curves on polystyrene using AM-AM, AM-PM, AM-FM configurations for the first mode (left) and second mode (right). The changes in effective cantilever stiffness for both modes (below) calculated from the observables of each mode (above) by using Equations 17–19, respectively (reference amplitude *A*_r1_ = 60 nm).

Figure 5 shows approach curves acquired in the AM-AM, AM-PM, and AM-FM configurations. Equations 17–19 were used to recover values of Δ*k*_1_ and Δ*k*_2_ where appropriate. The excellent agreement between all three bimodal configurations across the entire approach curve suggests that the simple harmonic oscillator is a valid model to describe the second eigenmode of the cantilever in this experiment.

### Calibration of the tip shape and size

When driving the first eigenmode in the AM mode, large variations in indentation depth can be achieved by varying the reference amplitude *A*_r1_ ("free amplitude") while keeping *A*_1_ fixed. Varying the reference amplitude is more effective than changing *A*_1_ which carries a weak dependence on indentation for a setpoint around *A*_1_/*A*_r1_ ≈ 0.5. Consecutive bimodal approach curves on a polystyrene sample were performed while varying the reference amplitude *A*_r1_ between 50 nm and 91 nm in 21 increments. The AM-AM, AM-PM, and AM-FM configurations were alternated. A total of 63 approach curves were performed within 45 min.

**Figure 6:**
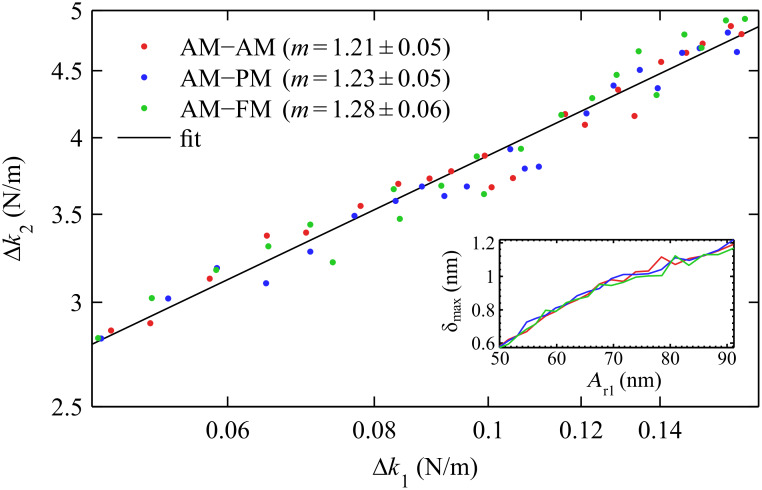
The set of approach curves in Figure 5 was repeated 21 times at various drive amplitudes. The values of Δ*k*_1_ and Δ*k*_2_ extracted at *A*_1_ = 40 nm from all 63 approach curves are plotted on a log–log scale. The line represents a power-law fit to the entire data, which can be used to determine the tip shape (*m* = 1.24) by Equation 16 and the tip size (*L* = 32 nm) by Equation 15. The inset shows the range of indentation depths probed in this experiment.

The values of Δ*k*_1_ and Δ*k*_2_ at a fixed amplitude (*A*_1_ = 40 nm) were used to determine the tip shape using the scaling law in Equation 16. The results are shown in Figure 6, where the exponent *m* values for each bimodal configuration were extracted from a power-law fit and shown to be equal within error; they averaged to *m* = 1.24 ± 0.04. This measurement of the tip shape (*m*) can now be used to calculate the tip size (*L*), which is uniquely determined if the sample modulus is known. Fitting Equation 15 to the data and setting *E*_eff_ = 3 GPa (as approximately expected for polystyrene [67]) results in a value for the tip length scale *L* = 32 nm. This tip characterization suggests a tip shaped as a rounded punch, somewhere between a punch of radius *R* = 32 nm and a paraboloid of radius *R =* 16 nm.

With a defined exponent *m* value, the absolute indentation depth at the various *A*_r1_ can be calculated by Equation 15, as shown in the inset of Figure 6. The calibration of the tip shape and size is expected to be valid for the explored range of indentations (between 0.6 nm and 1.2 nm in this case).

### Measurement of modulus

**Figure 7:**
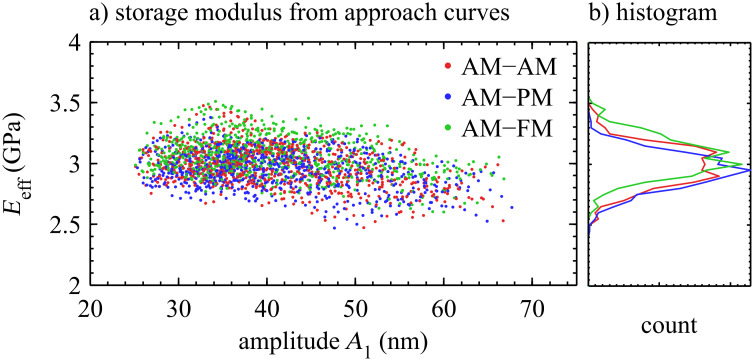
a) The modulus *E*_eff_ for all 63 approach curves from Figure 6 was extracted as a function of oscillation amplitude *A*_1_. Not only is *E*_eff_ independent of interaction amplitude *A*_1_ and reference amplitude *A*_r1_ (free amplitude), the three configurations (AM-AM, AM-PM, AM-FM) yield the same results within error. Note that a value of *E*_eff_ was only extracted for values *A*_1_/*A*_r1_ < 0.75 to ensure that repulsive interactions dominate.

With a modeled tip shape and size (*m* = 1.24, *L* = 32 nm), the modulus *E*_eff_ was extracted from all 63 approach curves, as shown in Figure 7. At *A*_1_ = 40 nm, the average modulus must be 3 GPa because this was the assumed value from which the tip size was extracted. Notably, the modulus appears to be independent of both *A*_1_ and bimodal configuration, as will be discussed in the next section.

## Discussion

### Independence of imaging parameters

The approach curves in Figure 7 span indentation depths from 0.6 nm to 1.2 nm, interaction amplitudes from 25 nm to 70 nm, and reference amplitudes between 50 nm and 91 nm. The modulus values at *A*_1_ = 40 nm are constrained to match 3 GPa by the tip shape and size calibration. However, throughout this range of imaging parameters, the modulus also remains within a 0.15 GPa standard deviation (5%) of 3 GPa, with no obvious trend. In addition, the three bimodal configurations agree to better than this standard deviation.

The independence on imaging parameters provides confidence that the system was accurately modeled by a rounded punch of shape *m* = 1.24 and size *L* = 32 nm. In contrast, assuming a paraboloidal tip (*m* = 1.5) results in increased variability in the modulus, as well as noticeable dependence on imaging parameters. Also, assuming a paraboloidal tip results in a tip radius *R* = 93 nm for these experiments, which is far from the nominal radius for this cantilever tip (*R* = 28 ± 10 nm).

These results lead to the conclusion that calibrating the tip shape and size provided a more accurate picture of the tip–sample contact mechanics, which led in turn to improved accuracy in extracting the modulus of polystyrene over a wider range of imaging parameters.

Given that the bimodal AFM theory was derived in the context of Hertzian contact mechanics where only repulsive interactions are considered, only data where *A*_1_/*A*_r1_ < 0.75 were analyzed to ensure that repulsive forces dominate over attractive and adhesive forces. This criterion is somewhat arbitrary and specific to the current dataset; other samples and tip geometries may require different threshold values of *A*_1_/*A*_r1_ or may require more elaborate models.

### AM/PM/FM equivalence

The agreement between AM, PM and FM modes of operation for Δ*k*_2_ shown in Figure 5 validates the mathematical framework leading up to Equations 17–19 that assumes SHO behavior of the cantilever. This agreement is reassuring, as a sinusoidally-driven SHO model was used for both eigenmodes to derive the bimodal interaction theory that relates the cantilever parameters to the interaction profile. Furthermore, the agreement between the modes of operation suggests that the feedback loops used for the PM and FM modes tracked changes in the cantilever eigenmode appropriately.

#### Large and small amplitude approximations

The amplitude of the first mode was modeled in the large limit, while the amplitude of the second mode was modeled in the small limit. The validity of these assumptions was implicitly verified in the dataset acquired for this experiment, as described herein.

The analysis in Figure 7 suggests that the variations in interaction amplitude (25 nm < *A*_1_ < 70 nm) do not affect the outcome of the measured *E*_eff_. This suggests that the large-amplitude limit for the first mode was fulfilled across the range of explored amplitudes.

The small-amplitude limit for *A*_2_ is tested by the comparison of experimental configurations. Whereas the second mode amplitude *A*_2_ for AM-AM drops significantly (from 500 pm to 200 pm) during a single approach curve, *A*_2_ for AM-PM and AM-FM is held fixed by the AGC. The fact that all three configurations lead to the same extracted *E*_eff_ suggests that the small-amplitude limit for the second mode was fulfilled.

#### Sensitivity of bimodal AFM

The high sensitivity of bimodal AFM was predicted in the theory described in Section ‘Bimodal interaction theory’ and attributed to the introduction of an additional eigenmode driven at a small amplitude. The argument was based on the fact that the weight function of the second eigenmode 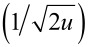 rises quickly as the tip approach the sample, as shown in Figure 3f.

This fact is demonstrated experimentally in the data of Figure 5, where Δ*k*_2_ is 20× to 50× larger than Δ*k*_1_. Whereas both Δ*k*_1_ and Δ*k*_2_ are weighted integrals of the identical stiffness profile *k*_int_(δ), the weight function of the second mode captures much more of the interaction stiffness for small indentations.

This illustrates the benefit of introducing a small-amplitude second eigenmode into the measurement: it provides a high-sensitivity channel of information, without disrupting the measurement as explained in the previous section.

#### Error from binomial approximation

The error introduced by the 
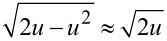
 approximation that allowed the analytical closed-form in Equation 14 and Equation 15 is now calculated. In the worst case scenario for this experiment with δ_max_ = 1.2 nm and *A*_1_ = 40 nm, a relative error of 0.5% is caused by the approximation 
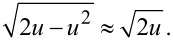
 In turn, this results in an overestimation of 0.9% in indentation depth, and an underestimation of 1.0% in modulus as far as Equation 14 and Equation 15 are concerned. These errors from approximation are negligible compared with experimental variability and absolute calibration error.

Importantly, applying Equation 19 to both eigenmodes results in FM-FM equations that are identical to those derived by Herruzo et al. [38–39]. This leads to the conclusion that the 
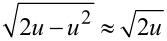
 approximation is sufficient for deriving analytical equations for bimodal FM-FM and other bimodal configurations. In other words, the use of fractional calculus, Laplace transforms, Padé approximants, and Bessel functions can be avoided with no loss in accuracy when deriving bimodal AFM theory.

#### Merits of AM-FM

Although the three configurations of bimodal methods tested in Figure 5 were equally accurate, the AM-AM method has the disadvantage of potentially having more noise relative to AM-PM, which in turn may have more noise relative to AM-FM. In AM-AM and AM-PM, the second eigenmode drive frequency is held fixed. In that case, when the second eigenmode resonance frequency shifts by more than its bandwidth (*f*_c_/2*Q*_c_), the signal-to-noise ratio degrades because the signal drops relative to the noise floor. Furthermore, in AM-AM operation, *A*_2_ decreases upon interaction with the surface – further aggravating the drop in signal-to-noise ratio. It is therefore often advisable to operate the second eigenmode in FM mode in practical situations.

On the other hand, the first eigenmode typically undergoes more modest changes in resonance frequency upon interaction with the surface. Also, these changes are controllable by the AFM user by the setting of an amplitude setpoint. For this reason, AM operation on the first eigenmode does not cause the same decrease in signal-to-noise ratio often observed on the second eigenmode. More importantly, dynamic AFM imaging in ambient conditions has relied on the robustness of AM (tapping-mode) imaging, mostly attributable to the monotonicity of the amplitude versus distance relationship, for stable operation and topography tracking.

For these two reasons, the authors generally recommend the use of AM-FM over the other configurations (AM-PM, FM-FM, etc.), as it is the most robust, versatile and sensitive configuration for bimodal nanomechanical mapping in a wide range of imaging conditions.

## Conclusion

An analysis framework for bimodal AFM with a power-law tip and Hertzian contact was presented. The derived theory was used to extract the tip shape and size for an experiment on a polystyrene sample. For a wide range of imaging parameters, the experimental data returned a nearly constant modulus of the material when analyzed with this model. Three configurations (AM-AM, AM-PM, AM-FM) were tested and shown to provide equally accurate results, thereby supporting our assumption that the cantilever second eigenmode can be modeled as a simple harmonic oscillator for the range of interactions explored in the experiment.

The approximations used for deriving an analytical closed-form solution for bimodal AFM were also investigated. Both the first mode large-amplitude approximation and the second mode small-amplitude approximation were verified to be accurate for typical imaging conditions. Notably, the fact that the weight function of the second eigenmode increases drastically as the tip approaches the sample explains the high sensitivity and spatial resolution of bimodal imaging. The binomial approximation of the stiffness weight function 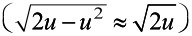 was shown to introduce negligible error (<1%), yet it can be used to derive bimodal AFM theory without invoking the use of fractional calculus.

The experimental in situ determination of the tip shape and size is a pivotal step towards absolute quantitative nanomechanical measurements in a variety of techniques. This work demonstrates the benefits of tip characterization in the context of bimodal nanomechanical mapping, which improves the accuracy of fast parametric techniques such as AM-FM nanomechanical mapping.

## Appendix

### a. Correction factor for contact radius: α_c_

The correction factor that accounts for deformation of the sample that leads to a true contact radius different from the nominal contact radius [68–69] is given by

[20]
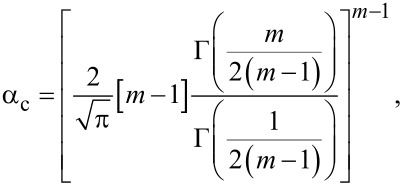


where Γ is the gamma function. Figure 1 graphs α_c_ for values between *m* = 1 and *m* = 2. Note that obtaining α_c_ = 1 at the value at *m* = 1 requires a taking a limit.

### b. Weighted interaction stiffness of the higher eigenmode

The weighted integral used to average the interaction stiffness across one oscillation cycle of the first eigenmode (Equation 6) also applies to higher eigenmodes; it can be rewritten as

[21]
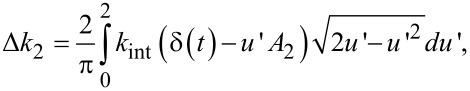


where *u*’ is the normalized deflection of the higher eigenmode in this context. The major distinction between Equation 6 and Equation 21 is the substitution δ_max_ → δ(*t*) which accounts for the non-zero amplitude of the first eigenmode *A*_1_ that introduces a time-varying component to the *k*_int_ experienced by the higher mode. For a first-mode period *T* = 1/*f*_c1_, this effect can be averaged over a full cycle by

[22]
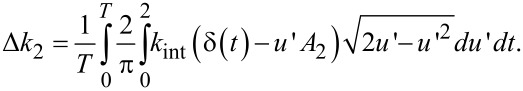


In the limit that *A*_2_ → 0 and *f*_c2_/*f*_c1_ → ∞, *k*_int_ remains constant throughout any full oscillation cycle of the second mode, such that Equation 22 solves to

[23]
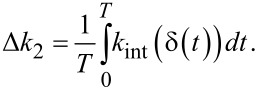


Notably, this integral that determines Δ*k*_2_ only depends on the trajectory of the first eigenmode because *A*_2_ is assumed small.

### c. Correction factor for power-law force model: β

When integrating *k*_int_(δ) in Equation 6 and Equation 9 to obtain Δ*k*_1_ and Δ*k*_2_, respectively, for a power-law model 
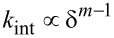
 as defined by Equation 5, gamma functions emerge due to the integration of non-integer powers. They are summarized here as

[24]
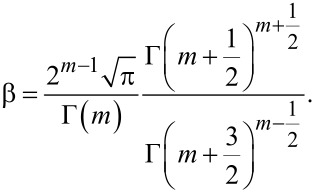


Note that the gamma functions that compose α_c_ in Appendix a relate to deformation of the sample and have no direct relationship to the gamma functions of β, which relate to the effects of power-law stiffness profiles affecting the cantilever parameters.

### d. Generalized SHO equation

The on-resonance Equations 17–19 are special cases of the general solution to a simple harmonic oscillator model, which can be driven on or off resonance with concurrent changes in drive frequency, phase, oscillation amplitude and drive amplitude. This section derives a general equation that makes no assumption about which of these variables is held fixed upon interaction with the sample.

The response of a freely vibrating cantilever with effective stiffness *k*_c_, mass *m*_c_, and damping *b*_c_ can be described by the complex-valued cantilever impedance [70]

[25]
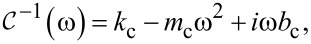


where the angular frequency is defined as ω = 2π*f*. The subscript “c” reminds that *k*_c_, *m*_c_, *b*_c_ are properties of the cantilever (prior to the tip–sample interaction) that do not change throughout the experiment.

A “reference” measurement of this cantilever impedance can be made prior to tip–sample interaction by exciting the cantilever sinusoidally with some reference driving force *F*_r_ at some reference drive frequency ω_r_, which results in a reference oscillation amplitude *A*_r_ and a reference phase 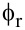:

[26]
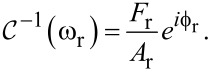


The reference driving force can be defined by isolating the imaginary components of both equations and solving for the driving force:

[27]
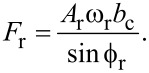


In the presence of some interaction, the cantilever impedance is subject to a time-averaged change in stiffness Δ*k* and a time-averaged change in damping Δ*b*, such that the interaction impedance

[28]
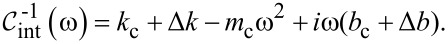


The interaction impedance is inferred during the experiment by

[29]
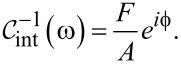


where the drive force *F*, the drive frequency ω, the oscillation amplitude *A* and the phase response 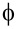 are measured during tip–sample interaction.

Subtracting the reference measurement 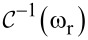 from the interaction measurement 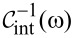, then isolating the real components and solving for Δ*k* leads to

[30]
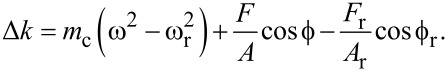


Assuming that the drive force *F* is proportional to the drive amplitude *D* and that their relationship is frequency-independent implies that

[31]
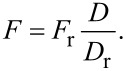


Substituting Equation 27 and Equation 31 into Equation 30 results in

[32]
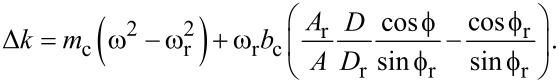


With the substitutions

[33]
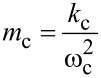


and

[34]
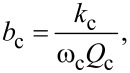


that introduce the cantilever resonance frequency ω_c_ and the quality factor *Q*_c_, Equation 32 can be rewritten in a more experimentally friendly form as

[35]
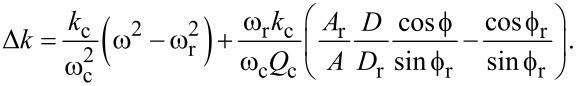


Lastly, driving the cantilever on resonance prior to tip sample interactions (ω_r_ = ω_c_; 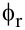 = 90°) simplifies the result to

[36]
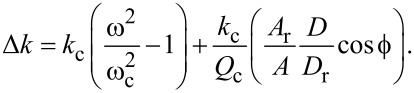


For AM operation, setting *D* = *D*_r_ and ω = ω_c_ results in Equation 17.

For PM operation, setting *A* = *A*_r_ and ω = ω_c_ results in Equation 18.

For FM operation, setting *A* = *A*_r_, enforcing 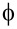 = 90°, and applying a binomial approximation results in Equation 19.
